# Novel HIV-1 Recombinant Forms in Antenatal Cohort, Montreal, Quebec, Canada

**DOI:** 10.3201/eid1702.100629

**Published:** 2011-02

**Authors:** Mathieu Quesnel-Vallières, Iman Kouzayha, Evelyne Tran, Issatou Barry, Charlène Lasgi, Natacha Merindol, Vanessa Monteil, Doris G. Ransy, Marc Boucher, Normand Lapointe, Hugo Soudeyns

**Affiliations:** Author affiliations: Centre Hospitalier Universitaire Sainte-Justine, Montreal, Quebec, Canada (M. Quesnel-Vallières, I. Kouzayha, E. Tran, I. Barry, C. Lasgi, N. Merindol, V. Monteil, D.G. Ransy, M. Boucher, N. Lapointe, H. Soudeyns);; Université de Montréal, Montreal (M. Quesnel-Vallières, I. Kouzayha, E. Tran, I. Barry, N. Merindol, D.G. Ransy, M. Boucher, N. Lapointe, H. Soudeyns);; Université Pierre et Marie Curie, Paris, France (C. Lasgi)

**Keywords:** HIV-1, AIDS, genetic recombination, viral subtypes, pregnancy, Canada, viruses, expedited, dispatch

## Abstract

Near full-length genomes of 4 unclassified HIV-1 variants infecting patients enrolled in an antenatal cohort in Canada were obtained by sequencing. All 4 variants showed original recombination profiles, including A1/A2/J, A1/D, and A1/G/J/CRF11_cpx structures. Identification of these variants highlights the growing prevalence of unique recombinant forms of HIV-1 in North America.

HIV-1 displays extensive genetic diversity. Group M includes 9 subtypes and >45 circulating recombinant forms (CRFs) (*1*). In western and central Africa, where the highest levels of HIV-1 genetic heterogeneity are observed, most subtypes cocirculate along with CRFs and unique recombinant forms (URFs). This diversity may complicate diagnosis and treatment of HIV infection and represents a challenge for vaccine design. In North America, the HIV-1 epidemic is dominated by subtype B; non-B subtypes are infrequently reported (*2**,**3*). Nonetheless, recent studies have shown a growing prevalence of non-B variants (*4**,**5*). In 2005, Akouamba et al. reported high levels of HIV-1 genetic diversity among participants in the Centre Maternel et Infantile sur le SIDA (CMIS) antenatal cohort of Centre Hospitalier Universitaire (CHU) Sainte-Justine, Montreal, Canada (*6*). Of these patients, 44 of 103 were infected with non-B subtypes, including 4 variants that failed to group within known subtypes in phylogenetic analyses (*6*). We performed near full-length genomic sequencing to characterize these 4 unassigned variants.

## The Study

All 4 patients were newcomers to Canada from sub-Saharan Africa who received prenatal care at CMIS during 1999–2003 (*6*). Patient TV721 emigrated from the Democratic Republic of Congo, TV749 from Congo, and TV725 and TV919 from Rwanda. HIV-1 viral loads at study entry, measured by the Versant HIV-1 RNA 3.0 assay (bDNA) (Bayer, Pittsburgh, PA, USA), with a limit of detection of 50 RNA copies/mL plasma, were 164–23,369 RNA copies/mL plasma. CD4+ T cell counts ranged from198 cells/mm^3^ to 816 cells/mm^3^ (Table). Standardized clinical follow-up, including antiretroviral prophylaxis and treatment, was provided to all women and their children. This study was conducted according to the guidelines of the Ethics Review Board of CHU Sainte-Justine.

**Table:** Virologic and immunologic parameters in patients participating in antenatal cohort, Canada*

Patient	Age, y	CD4 count, cells/mm^3^	Viral load, RNA copies/mL	Antiretroviral treatment
TV721	33.2	210	164	AZT-3TC-NVP
TV725	30.1	816	739	None
TV749	34.1	198	23,369	AZT-3TC-NFV
TV919	34.0	420	1,910	AZT-3TC-NFV

*AZT, zidovudine; 3TC, lamivudine; NVP, nevirapine; NFV, nelfinavir.

Viral RNA was extracted from serum and amplified by using custom-designed primers and the QIAGEN OneStep reverse transcription–PCR (QIAGEN, Mississauga, ON, Canada) (sequences available on request). For each isolate, 14–20 amplicons spanning the complete genome were generated and subcloned into pCR 2.1 TOPO (Invitrogen, Carlsbad, CA, USA). For each amplicon, we sequenced 3–10 independent clones (Beckman-Coulter, Palo Alto, CA, USA). Chromatograms were edited with Chromas version 1.45 (Technelysium, Southport, Queensland, Australia). Overlapping segments were aligned by using ClustalX version 1.81 (*7*) and assembled manually. Consensus sequences were generated by selecting the most frequent nucleotide at each position. We performed bootscan analyses according to the neighbor-joining method and Kimura 2-parameter distances using a 300-nt window and 10-nt increments (Simplot 3.5.1) (*8*). These parameters allow accurate localization of recombination breakpoints in HIV-1 recombinants (*9*). We computed phylogenetic reconstructions based on the neighbor-joining method and Kimura 2-parameter distances by using MEGA4 (*10*) to confirm recombinant structures. Bootstrap values >80% were considered significant.

Complete HIV-1 genomic sequences were obtained from patients TV721 (9,794 nt) and TV749 (9,791 nt). Genomic coverage of 79.3% and 91.6% was achieved for patients TV725 (7,763 nt) and TV919 (8,905 nt), respectively. On the basis of HXB2 numbering (*1*), missing regions were located between positions 545–1411 and between 6946–7930 for patient TV725, and between positions 7138 and 7834 for patient TV919. Screening of HIV-1 genomic sequences from patient TV721 with the HIVdb Genotypic Resistance Interpretation Algorithm (http://hivdb6.stanford.edu/asi/deployed/HIVdb.html) showed minor resistance mutations to protease inhibitors (L10I) and integrase inhibitors (I203M). Mutations associated with minor resistance to protease inhibitors (L10I) and non-nucleoside reverse transcription inhibitors (E138A) were detected in sequences from patient TV919, and a mutation conferring high-level resistance to delavirdine (P236L) was detected in patient TV725. In contrast, sequences from patient TV749 did not show mutations associated with resistance to antiretroviral agents (*11*). Previous subtyping, based on phylogenetic analyses of *pol* gene sequences, showed that sequences from patients TV721 and TV749 grouped together (100% bootstrap) but only loosely with clade J references (61% bootstrap); sequences from patients TV725 and TV919 grouped outside all major clades (*6*). Bootscan analysis showed complex recombinant structures for all 4 full-length or near full-length genomes (Figure). Sequences derived from patients TV721 and TV749 comprised regions from subtype J but were also similar to subtypes A1 and A2 (Figure). Examination of the homology, position, and sharing of recombination breakpoints suggests that HIV-1 isolates infecting patients TV721 and TV749 may be closely related recombinants, perhaps resulting from common-source transmission or representing a novel CRF or URF. Reciprocal bootscan analyses that included subtypes A1, A2, and J supported this assessment (data not shown). However, a review of the medical files and case histories of patients TV721 and TV749 did not confirm epidemiologic relatedness. Although multiple CRFs and URFs contain segments related to subtypes A and J, sequences from patients TV721 and TV749 are 2 of only 3 full-length HIV-1 genomes reported that exclusively comprise sequences related to subtypes A and J (*12*). The genomic structure of 98BW21.17 resembles that of sequences from patients TV721 and TV749 in terms of chimerism, but the location of recombination breakpoints is distinct (data not shown).

**Figure:**
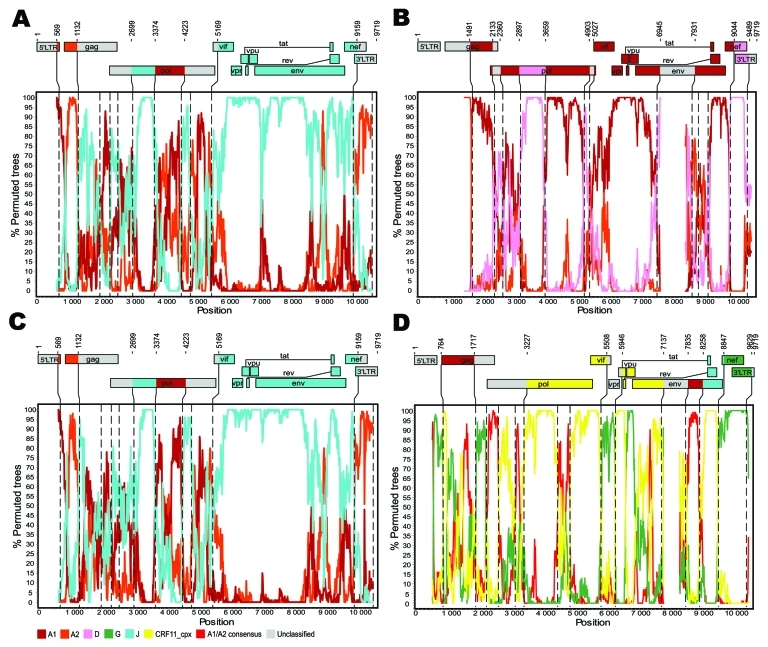
Genetic organization and recombination breakpoints in HIV-1 genomic sequences isolated from patients TV721 (A), TV725 (B), TV749 (C), and TV919 (D). Nucleotide sequences were submitted to GenBank (accession nos. HM215249–HM215252). Similarity plots were produced with Simplot version 3.5.1 (http://sray.med.som.jhmi.edu/SCRoftware/simplot) by using windows of 500 nt and increments of 50 nt to guide the choice of reference sequences used for bootscanning (*8*). Bootscan analyses were then performed according to the neighbor-joining method and Kimura 2-parameter distances. The size of the sliding window was set at 300 nt with 10-nt increments (*9*). Reference sequences used were subtype A1: A1.AU.03 (DQ676872), A1.KE.94 (AF004885), A1.RW.92 (AB253421); subtype A2: A2.CD.97 (AF286238), A2.CY.94 (AF286237); subtype D: D.CD.83.ELI (K03454), D.CM.01 (AY371157), D.TZ.01 (AY253311); subtype G: G.BE.96 (AF084936), G.KE.93 (AF061641), G.NG.92 (U88826); subtype J: J.CD.97 (EF614151), J.SE.93 (AF082394), J.SE.94 (AF082395); and CRF11_cpx: 11_cpx.CM.95 (AF492624). Phylogenetic reconstructions based on the neighbor-joining method and the Kimura 2-parameter distance model were computed by MEGA4 (*10*) and used to confirm the structures of the recombinants. Bootstrap values >80% (500 replicates) were considered significant. Vertical dashed lines indicate the position of recombination breakpoints. Numbering of residues is based on the sequence of HIV-1 HXB2 (GenBank accession no. K03455).

Sequences from patients TV725 and TV919 grouped outside all major clades in phylogenetic analyses of *pol* gene sequences (*6*). Bootscan analysis of the HIV-1 strain infecting patient TV725 clearly demonstrated that this variant comprised segments most closely related to subtypes A1 and D (Figure). Sequences from patient TV725 display a recombination pattern resembling that of CRF35_AD, the only other A1/D intersubtype recombinant described, which was recently identified in Kabul, Afghanistan (*13*). CRF35_AD and TV725 share A1 backbones, and recombination points bordering clade D segments are comparatively close (positions 2166–2444 for CRF35_AD and 2133–2360 for the isolate infecting patient TV725; positions 2901–3538 for CFR35_AD and 2897–3659 for TV725). However, they differ with respect to a clade D–related segment at positions 9044–9489 in TV725 (Figure). Finally, analysis of TV919 sequences showed complex A1/G/J mosaicism and similarities with CRF11_cpx, in terms of clade composition and localization of recombination breakpoints (*14*). Including the CRF11_cpx reference sequence 95CM1816 in bootscan analysis highlighted the similarities between sequences from patient TV919 and CRF11_cpx (Figure), which extends from positions 3227 to 7137 and includes segments corresponding to subtypes A1, G, and J. The most distinctive difference between the isolate infecting patient TV919 and CRF11_cpx started at position 7835; the former sequentially clustered with subtypes A1, J, and G, CRF11_cpx closely associated with subtypes J, A1, and E. Thus, the isolate infecting patient TV919 represents a novel A/G/J/CRF11_cpx recombinant. The HIV-1 isolates that infected patients TV721, TV725, TV749, and TV919 had more recombination breakpoints and unclassified regions than most CRFs and URFs, highlighting their unique recombination profiles and structural complexity.

## Conclusions

We identified novel HIV-1 recombinants infecting pregnant women in Montreal. None of the 4 patients transmitted HIV-1 to their children. No evidence was found that these particular variants currently circulate within the Canadian population. Thus, the HIV-1 isolates infecting patients TV721, TV725, TV749, and TV919 must be construed as URFs. In North America, only 1 URF, also isolated in Montreal, was characterized by near full-length genomic sequencing (*5*). Given the ongoing movement of the population from areas where the disease is endemic into regions in which subtype B predominates, reports of novel HIV-1 recombinants are likely to increase and include complex mosaic genomes. Biological properties of recombinant subtypes might differ from those of other clades, particularly in terms of HIV disease progression (*15*) and drug resistance. In terms of public health, antenatal cohorts represent unique sentinel sites to monitor the emergence of novel HIV-1 variants, including complex mosaic recombinants, in countries where their prevalence is low.
